# iTRAQ-based quantitative proteomic analysis of embryonic developmental stages in Amur sturgeon, *Acipenser schrenckii*

**DOI:** 10.1038/s41598-018-24562-1

**Published:** 2018-04-19

**Authors:** Shubo Jin, Dajiang Sun, Dan Song, Nianmin Wang, Hongtuo Fu, Feng Ji, Ying Zhang

**Affiliations:** 1 Heilongjiang River Fisheries Research Institute, Chinese Academy of Fishery Sciences, Haebin, China; 2 Key Laboratory of Freshwater Fisheries and Germplasm Resources Utilization, Ministry of Agriculture, Freshwater Fisheries Research Center, Chinese Academy of Fishery Sciences, Wuxi, China

## Abstract

The Amur sturgeon, *Acipenser schrenckii*, is an important aquaculture species in China with annual production of about 150 thousand tons in 2015. In this study, we investigated the regulatory proteins and pathways affecting embryonic development of Amur sturgeon, by analyzing of the differential proteomes among four embryonic developmental stages using isobaric tags for relative and absolute quantitation (iTRAQ), combined with the analysis of effects of microelements and antioxidants on embryonic development. Seventy-four, 77, and 76 proteins were differentially expressed according to iTRAQ analysis between the fertilized egg and blastula, blastula and neurula, and neurula and heart-beat stages, respectively. GO and KEGG enrichment analyses indicated that Gluconeogenesis, Ribosome and Proteasome were the most enriched pathways, which may promote energy formation, immune system protection and protein synthesis process in *A. schrenckii*. The measurement of microelements indicated that Mn, Cu and Fe were obtained from their parents or water environment in *A. schrenckii*, while Zn plays vital roles throughout embryonic development. The dramatically high level of malondialdehyde (MDA) across the embryonic development may be the main reason leading to a low hatching rate in *A. schrenckii*. This study provides the basis for further proteome analysis of embryonic development in *A*. *schrenckii*.

## Introduction

The Amur sturgeon (*Acipenser schrenckii*) is referred to as a ‘living fossil’, and is among the most primitive Actinopterygii species. It is distributed in the Amur, Songhua, and Heilong Rivers^1^. Amur sturgeon demonstrate a high growth rate and strong disease resistance, and is the most popular sturgeon aquaculture species in China with annual production of about 150 thousand tons in 2015, accounting for about 15% of the total sturgeon aquaculture production^2^. Several countries have recently shown an interest in Amur sturgeon aquaculture, and this species is now raised widely^3^. However, a low hatching rate represent the main problem affecting the sustainable development of the sturgeon aquaculture industry, especially for that at blastula stage, according to the hatching experience in our laboratory. A full understanding of the regulatory proteins and pathways involved in the embryonic development of *A. schrenckii*, and the effects of microelements and antioxidants on the embryonic development of *A. schrenckii* is therefore urgently needed. Several microRNA libraries and mRNA transcriptomes have been constructed for *Acipenser* species, and sex-related and reproduction-related genes have been investigated^4–8^. However, to the best of our knowledge, no previous studies have analyzed the changes in protein expression in this species.

The embryonic development of *A. schrenckii* can be divided into nine main stages: fertilized egg, cleavage stage, blastula, gastrula, yolk plug, neurula, formation of the optic bud, heart-beat stage, and hatching stages^9^. Among these, the fertilized egg, blastula, neurula, and heart-beat stages are particularly important stages of embryogenesis; the blastula stage indicates successful fertilization, while the neurula and heart-beat stages represent important steps in organogenesis, especially in relation to the heart and brain^9^.

Isobaric tag for relative and absolute quantization (iTRAQ) profiling is a reliable and accurate method of protein measurement, which is especially useful in annotated-related species. iTRAQ uses a variety of isotope reagents to label at the N-terminal of the protein peptide or the groups of lysine side chain, and can be used to examine proteomic profiles simultaneously between eight different samples^10^ via a high precision mass spectrometry^11^. Only small amount of protein was used for quantification. This new approach avoids some of the drawbacks of 2-DE, including identification of relative less number of proteins, difficulties in quantifying relatively small amounts of protein, and relatively complicated procedures^12^. iTRAQ has thus been used increasingly for proteomics analysis in recent years^12–17^.

The microelements are essential prosthetic groups and enzyme activators for aquatic animals, dramatically affecting the formation of bones, the synthesis of hormone and enzyme, the maintaining of health and normal growth, and the enhancing of the immune system^18^. Antioxidants has important regulatory roles in converting peroxides into harmful or harmless substances in the body through redox action. They immediately work once peroxides are formed in the body^19^.

In this study, we aimed to analyze changes in proteome profiles across embryonic development of *A. schrenckii*, and release the effects of microelements and antioxidants on *A. schrenckii* embryonic development. iTRAQ was used to analyze changes in proteome profiles during the fertilized egg, blastula, neurula, and heart-beat stages of embryonic development in *A. schrenckii*. We also investigated changes in levels of four microelements and four antioxidants during these stages. The combined results regarding changes in protein expression, microelements, and antioxidants will provide valuable information on the regulatory proteins and pathways affecting *A. schrenckii* embryonic development.

## Results

### iTRAQ quantification

We analyzed the tandem mass spectrometry (MS/MS) data using Mascot software. iTRAQ analysis of the proteomes of the four developmental stages of Amur sturgeon showed 217,935 spectra, including 3,142 identified peptides containing 2,929 unique peptides, and including 6,062 unique spectra, resulting in 1,143 protein hits.

### Protein mass, length of identified peptides, and peptide numbers of unique spectra

**Figure 1:**
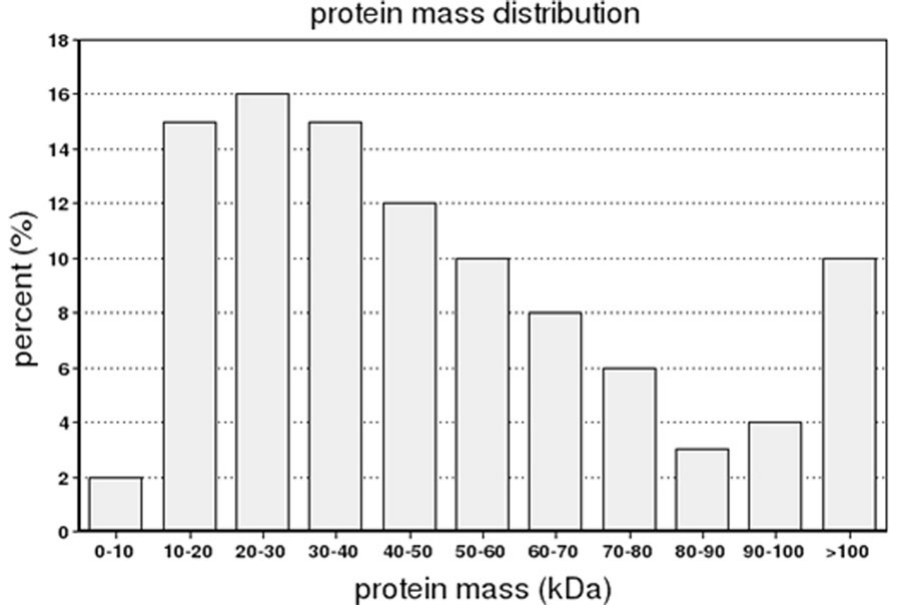
Protein mass distribution of Amur sturgeon peptides.

Most of the proteins were 20–30 kDa (16%) in protein mass, followed by 10–20 kDa (15%), and 30–40 kDa (15%) (Fig. 1), while 10% of peptides were >100 kDa.

The lengths of the 3,142 identified peptides ranged from 5–40 amino acids (aa). Most were 11 aa in length, followed by 12 and 10 aa, with almost 90% being 7–17 aa in length.

The peptide number of over 97% unique proteins was less than 10, in which 51.88% unique proteins only contain one peptide. The number of proteins decreased with the increase of peptide number.

### Protein function annotation

**Figure 2:**
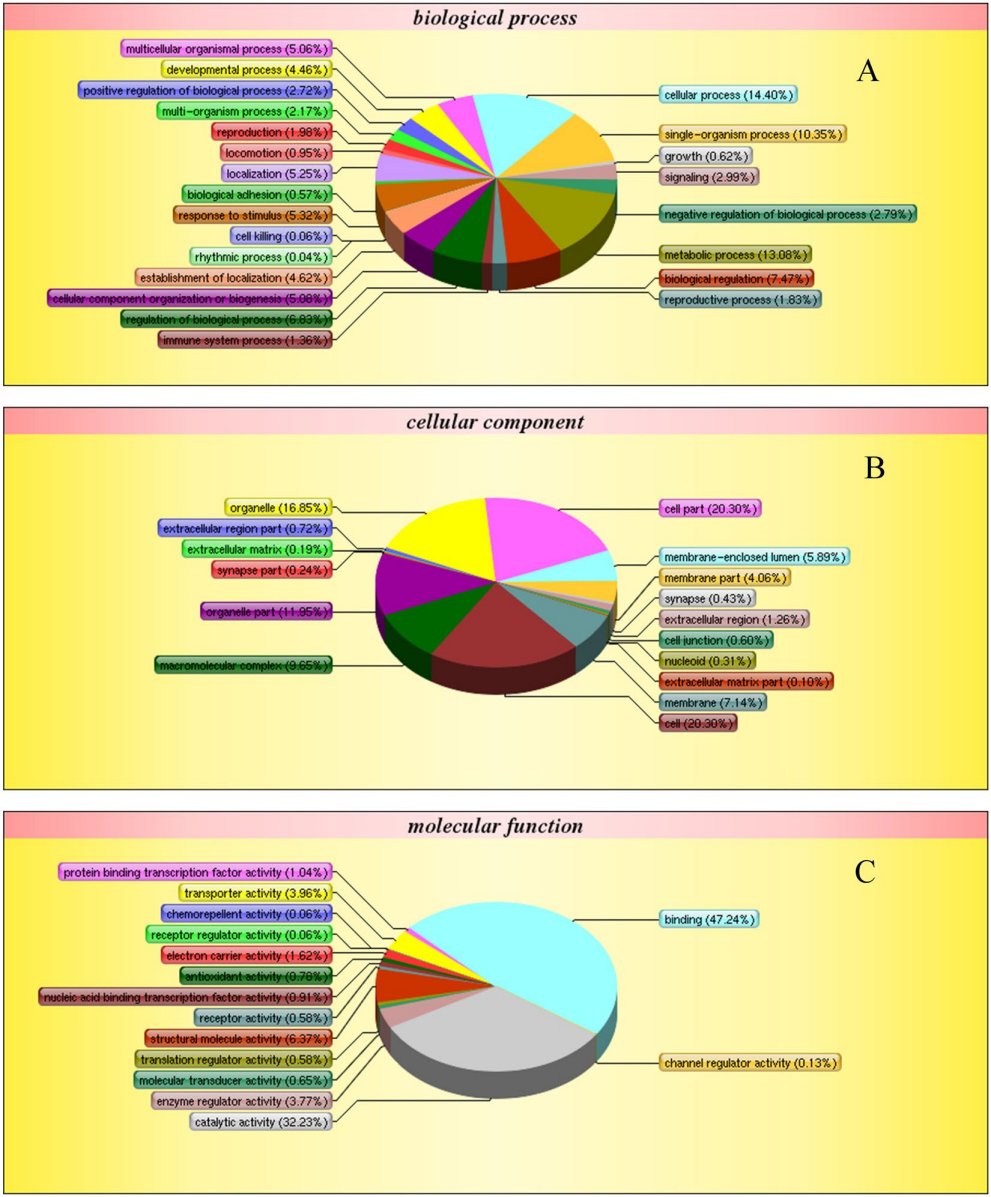
Gene ontology (GO) analysis of all proteins during the embryonic development identified by iTRAQ analysis. The left y-axis indicates the percentage of a specific category of genes existed in the main category, whereas the right y-axis indicates the number of a specific category of genes existed in main category. Shown above is the classification of these proteins in different categories based on biological process (**A**), cellular component (**B**) and molecular function (**C**).

The Gene Ontology (GO) and Clusters of Orthologous Groups (COG) tools aim to provide a structured and controlled vocabulary for describing gene products. GO includes three ontologies: cellular component, biological process, and molecular function. GO terms were assigned to 1,005 proteins based on BLAST matches with proteins with known functions. The matched proteins comprised 54 functional groups. The number of proteins in each GO term ranged from 1–841. Biological process mainly included cellular process (785, 14.40%), metabolic process (713, 13.08%), and single-organism process (564, 10.35%) (Fig. 2A); cellular component included cell part (841, 20.30%), cell (841, 20.30%), organelle (698, 16.85%), and organelle part (495, 11.95%) (Fig. 2B); and molecular function included binding (727, 47.24%) and catalytic activity (496, 32.23%) (Fig. 2C).

**Figure 3:**
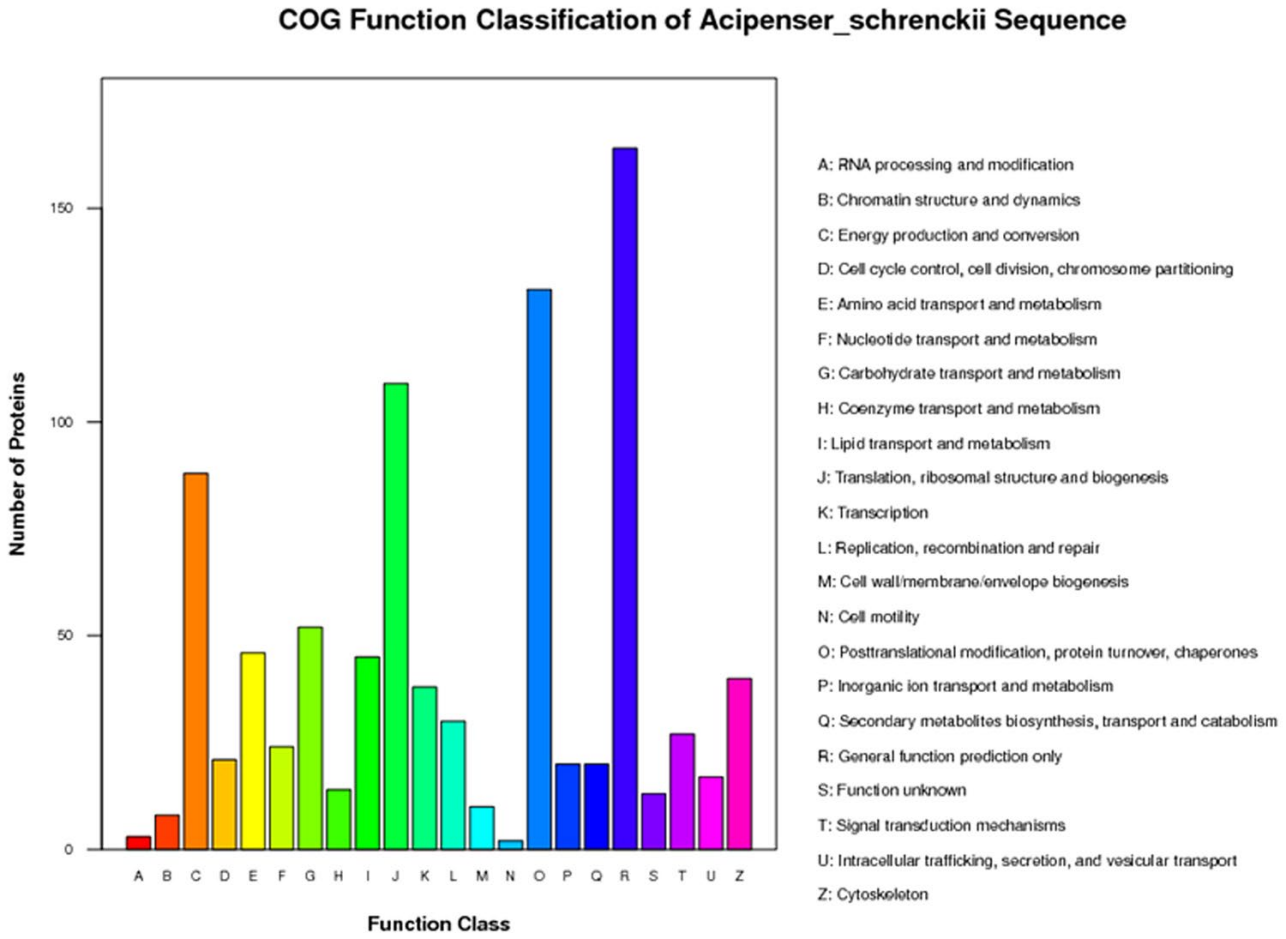
Cluster of orthologous groups (COG) classification of peptides. The peptides were classified functionally into 22 molecular families in the COG database.

COG annotation analysis classified the proteins identified by iTRAQ analysis into 22 categories (Fig. 3), with 2–164 proteins in each category. Among them, the general function prediction represented the largest functional group, including 164 proteins, followed by Posttranslational modification, protein turnover, chaperones (131), Translation, ribosomal structure and biogenesis (109), and Energy production and conversion (88). Cell motility, RNA processing and modification, and Chromatin structure and dynamics represented the smallest functional categories.

Kyoto Encyclopedia of Genes and Genomes (KEGG) (http://www.genome.jp/kegg) ontology assignments were used to classify functional annotations of the identified proteins to clarify their biological functions. Among the 1143 proteins, 981 proteins were identified in KEGG database based on BLAST matches with proteins with known functions, and mapped to 226 pathways. Metabolic pathway (226, 23.04%), Parkinson’s disease (64, 6.52%), Huntington’s disease (63, 6.42%), and Ribosome (62, 6.32%) represented the main metabolic pathways.

### Identification of differentially expressed proteins (DEPs)

According to previous studies, DEPs were defined based on a threshold 1.2–1.5-fold change^20–23^. In the current study, only proteins expressed in the four embryonic developmental stages with fold changes ≥1.2 or ≤0.83 and with P < 0.05 and FDR < 0.05 (one-way ANOVA) compared with their control groups were considered to be DEPs^12^. Seventy-four proteins were differentially expressed in the fertilized egg relative to the blastula stage, including 53 up-regulated and 21 down-regulated proteins (Supplementary information 1); 77 proteins were differentially expressed in the blastula relative to the neurula stage, including 37 up-regulated and 40 down-regulated proteins (Supplementary information 2); and 76 proteins were differentially expressed in the neurula relative to the heart-beat stage, including 25 up-regulated and 51 down-regulated proteins (Supplementary information 3). Among these DEPs, 31 were considered as “intersection DEPs”, which were differentially expressed in at least two of the comparisons (Table 1).

**Table 1:** List of intersection differentially expressed proteins (intersection DEPs) in *A. schrenckii* proteome. Intersection DEPs were identified as differentially expressed in at least two of the comparisons. FS: Fertilized egg stage. BS: Blastula stage. NS: Neurula stage. HB: Heart-beating stage.

Protein Name	Accession Number	P value	FSvsBS fold changes	BSvsNS fold changes	NSvsHB fold changes
ZP4 protein	gb|ABQ09466.1|	1.00E-56	1.72		1.319
ZP2 protein	emb|CAA96573.1|	1.00E-20	1.42		1.282
60S ribosomal protein l7	gb|ADO28714.1|	6.00E-87	0.64		0.812
Zona pellucida sperm-binding protein 4	sp|Q12836|	1.00E-30	1.67	0.82	
26S protease regulatory subunit 6A	ref|NP_001134065.1|	0	1.259	0.776	
DNA helicase B	gb|EKC24286.1|	3.00E-19	1.77	2.398	
Probable serine carboxypeptidase	emb|CAG12003.1|	1.00E-06	1.65		0.428
Zona pellucida glycoprotein	gb|AEQ59107.1|	1.00E-40	2.0		0.829
Hydroxysteroid dehydrogenase-like protein 2	ref|NP_001135155.1|	1.00E-67	1.67		0.612
Zona pellucida glycoprotein 3	gb|ACO54852.1|	8.00E-162	1.60		1.75
Aldehyde dehydrogenase family 9 member A1-A	ref|XP_003444296.1|	0	0.46	1.664	
T-complex protein 1 subunit alpha	gb|AAA40337.1|	1.00E-116	0.56	1.245	
NAD binding	ref|XP_003963232.1	2.00E-170	0.636	0.387	
Peroxiredoxin-4	gb|EMP25650.1|	2.00E-88	0.79	1.211	
Keratin, type I cytoskeletal 50 kDa	emb|CAD20811.1|	2.00e-59	0.62	0.303	0.53
Keratin, type II cytoskeletal 8	emb|CAD38126.1|	1.00E-32	0.514		0.584
Myosin regulatory light chain 2	dbj|BAB69824.1|	3.00E-86	0.611		0.384
Uncharacterized methyltransferase ydaC-like	ref|XP_003448322.1|	4.00E-68	0.69	1.462	
Hypothetical protein EGM_06981	gb|EHH57372.1|	3.00E-35	0.62	1.595	
Ribosomal protein L22	gb|ACH85303.1|	3.00E-39	0.48		0.696
Glyceraldehyde-3-phosphate dehydrogenase	gb|ABG56069.1|	9.00E-42	0.41		1.385
Zona pellucida sperm-binding protein 3-like	ref|XP_003451303.1|	1.00E-11	0.76		1.877
Cytochrome P450 2K1	gb|EMP34025.1|	5.00E-159	0.74	1.256	
Unnamed protein product	dbj|BAE90248.1|	9.00E-19		1.412	1.28
Cytoplasmic dynein 1 heavy chain 1	ref|XP_002825165.1|	0		1.486	0.616
meprin A alpha-like	ref|XP_002734255.1|	2.00E-23		1.603	6.606
heat shock protein 90 beta	gb|AFS88930.1|	0	0.80	0.823	
Histone H2A.x	ref|XP_003976990.1|	7.00E-51		0.449	0.592
60S ribosomal protein L12	gb|ACH70859.1|	7.00E-86		0.762	0.786
Keratin, type II cytoskeletal 8	emb|CAD38126.1|	1.00E-32		0.514	0.584
Myosin regulatory light chain 2	dbj|BAB69824.1|	3.00E-86		0.611	0.384

The main metabolic pathways of the DEPs are shown in Table 2. The main metabolic pathways enriched among these embryonic developmental stages included Glycolysis/Gluconeogenesis, Ribosome, Metabolic pathways, and Alzheimer’s disease, which were the main “intersection metabolic pathways” during embryonic development. Glyceraldehyde-3-phosphate dehydrogenase A (GAPDH) (fertilized egg vs blastula with fold change of 0.41, and neurula vs heart-beat stages with fold change of 1.39) and nicotinamide adenine dinucleotide (NAD)binding (fertilized egg vs blastula with fold change of 0.64, blastula vs neurula with fold change of 0.39) in Gluconeogenesis metabolic pathway, the 60S ribosomal proteins L12 (blastula vs neurula with fold change of 0.76, and neurula vs heart-beat stages with fold change of 0.79), L17 (fertilized egg vs blastula with fold change of 0.64, and neurula vs heart-beat stages with fold change of 0.81), and L22 (fertilized egg vs blastula with fold change of 0.48, and neurula vs heart-beat stages with fold change of 0.69) in Ribosome metabolic pathway, and 26S protease regulatory subunit 6A (fertilized egg vs blastula with fold change of 1.26, and blastula vs neurula with fold change of 0.78) in Proteasome were identified as “intersection DEPs”.

**Table 2:** Parts of KEGG enrichment pathway. FS: Fertilized egg stage. BS: Blastula stage. NS: Neurula stage. HB: Heart-beating stage.

Pathway (FSvsBS)	DEPs (60)	P value	Pathway (BSvsNS)	DEPs (71)	P value	Pathway (NSvsHB)	DEPs (67)	P value
Glycolysis/Gluconeogenesis	5 (8.33%)	0.06290401	Glycolysis/Gluconeogenesis	8 (11.27%)	0.002957551	Glycolysis/Gluconeogenesis	3 (4.48%)	0.4516816
Ribosome	4 (6.67%)	0.535082	Ribosome	8 (11.27%)	0.07119152	Ribosome	10 (14.93%)	0.006889619
Metabolic pathways	16 (26.67%)	0.2917374	Metabolic pathways	16 (22.54%)	0.5905762	Metabolic pathways	13 (19.4%)	0.8097841
Alzheimer’s disease	5 (8.33%)	0.2898660	Alzheimer’s disease	2 (2.82%)	0.9389918	Alzheimer’s disease	3 (4.48%)	0.7853702
Proteasome	4 (6.67%)	0.09527895	Proteasome	3 (4.23%)	0.351062	Citrate cycle (TCA cycle)	4 (5.97%)	0.1177314
Pathways in cancer	4 (6.67%)	0.1153441	Pathogenic Escherichia coli infection	5 (7.04%)	0.07474247	Hypertrophic cardiomyopathy (HCM)	4 (5.97%)	0.1061580
Epstein-Barr virus infection	4 (6.67%)	0.1371261	Epstein-Barr virus infection	5 (7.04%)	0.0832896	Dilated cardiomyopathy	4 (5.97%)	0.1177314
PPAR signaling pathway	3 (5%)	0.08014134	Phagosome	4 (5.63%)	0.08885731	Salmonella infection	4 (5.97%)	0.1177314
Glutathione metabolism	3 (5%)	0.09211395	Regulation of actin cytoskeleton	4 (5.63%)	0.2932850	Cardiac muscle contraction	4 (5.97%)	0.2268845
Herpes simplex infection	3 (5%)	0.1614924	Fructose and mannose metabolism	3 (4.23%)	0.03934877	Salivary secretion	3 (4.48%)	0.03382282
Valine, leucine and isoleucine degradation	3 (5%)	0.1927517	Propanoate metabolism	3 (4.23%)	0.1030570	Systemic lupus erythematosus	3 (4.48%)	0.06441405
Pathogenic Escherichia coli infection	3 (5%)	0.3107661	Pyruvate metabolism	3 (4.23%)	0.228632	Tight junction	3 (4.48%)	0.4140042
Protein processing in endoplasmic reticulum	3 (5%)	0.5132316	Oxidative phosphorylation	3 (4.23%)	0.7671755	Regulation of actin cytoskeleton	3 (4.48%)	0.4883963

### Microelement contents

The contents of Fe, Mn, and Cu decreased gradually from the fertilized egg to the neurula stage, and then increased again at the heart-beat stage. The Zn content fluctuated during the four stages. The peaks Fe, Mn, Cu, and Zn contents occurred at the fertilized egg, fertilized egg, heart-beat, and blastula stages, respectively (Table 3).

**Table 3:** The contents changes of microelements (mg/KG). Data are shown as mean ± SD (standard deviation) of each developmental stage in three separate individuals. Little letters indicate expression difference of microelements in different developmental stages.

Embryonic developmental stages	Fe	Mn	Cu	Zn
Fertilized egg	17.01 ± 0.36^a^	1.04 ± 0.02^a^	1.59 ± 0.04^a^	9.72 ± 0.18^a^
Blastula	14.63 ± 0.21^b^	0.79 ± 0.01^b^	1.46 ± 0.02^b^	10.33 ± 0.21^b^
Neurula	14.25 ± 0.01^c^	0.68 ± 0.01^c^	1.45 ± 0.01^b^	8.10 ± 0.09^c^
Heart-beat Stage	16.55 ± 0.55^a^	0.78 ± 0.02^b^	1.95 ± 0.04^c^	9.68 ± 0.13^a^

### Antioxidant contents

Superoxide dismutase (SOD), MDA, catalase (CAT), and glutathione (GSH) showed similar expression patterns during the four embryonic developmental stages. These four antioxidants increased from the fertilized egg to blastula stages, deceased to the neurula stages, and then increased significantly again to the heart-beat stage. The peak GSH, MDA, and SOD contents were observed at the heart-beat stage, while the peak CAT content was observed at the blastula stage (Table 4). The GSH and SOD levels at heart-beat stage are 10 times and 5 times higher than other stages (P < 0.01). The level of MDA at each developmental stage was hundred folds higher than that of other antioxidants (P < 0.01).

**Table 4:** The contents changes of antioxidants. Data are shown as mean ± SD (standard deviation) of each developmental stage in three separate individuals. Little letters indicate expression difference of antioxidants in different developmental stages.

Embryonic Developmental Stages	GSH (U/g protein)	MDA (U/mg protein)	SOD (U/g protein)	CAT (U/g protein)
Fertilized egg	33.45 ± 11.36^a^	11.02 ± 1.97^a^	64.48 ± 11.66^a^	8.18 ± 0.91^a^
Blastula	54.89 ± 13.29^b^	16.01 ± 2.91^b^	115.05 ± 21.71^b^	26.3 ± 6.48^b^
Neurula	43.63 ± 10.44^b^	8.3 ± 1.35^c^	55.84 ± 12.77^a^	11.65 ± 3.69^c^
Heart-beat Stage	451.21 ± 36.74^c^	17.39 ± 2.49^b^	256.03 ± 38.93^c^	22.29 ± 5.01^b^

## Discussion

This study provides valuable evidence for proteomic changes during embryonic development in *A. schrenckii* based on iTRAQ profiling. To the best of our knowledge, this is the first proteome-profiling analysis in *A. schrenckii*, and the results will thus promote further studies to improve our understanding of the regulatory proteins and pathways affecting embryonic development in this species. A total of 1,143 proteins were identified, mostly with biological functions related to Cell part, Cell, Cellular process, Binding, and Metabolic process according to GO assignments, and General function prediction only, Posttranslational modification, protein turnover, chaperones, and Translation, ribosomal structure, and biogenesis in COG analysis. Dramatically more than 1,134 proteins in total were identified by GO assignment and COG analysis, because several sequences were involved in more than one functional category. KEGG analysis can help to detect relationships between different genes. The main KEGG pathways included metabolic pathway, Parkinson’s disease, Huntington’s disease, and Ribosome.

**Figure 4:**
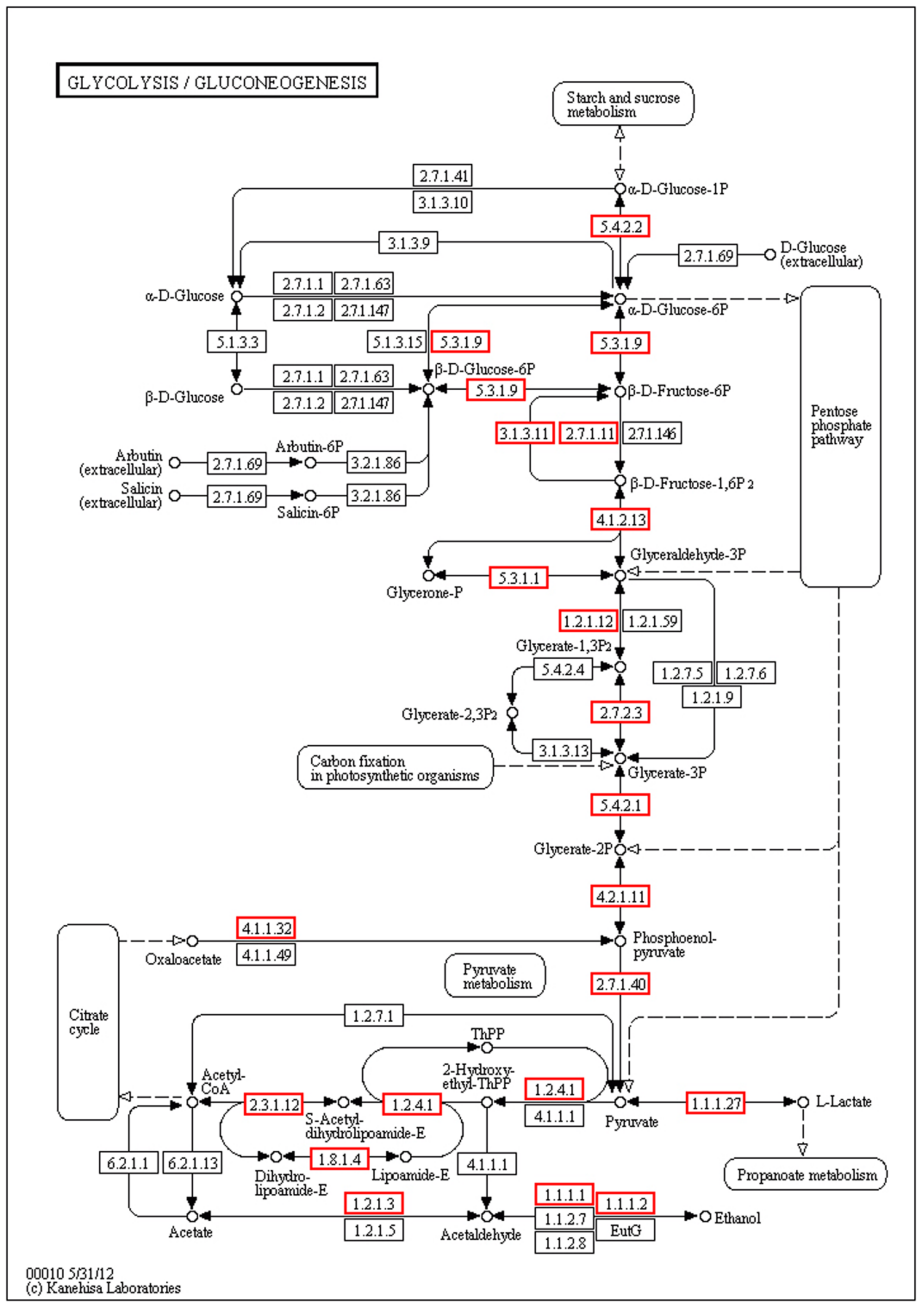
Glycolysis/Gluconeogenesis metabolic pathway. According to the KEGG analysis of DEPs, Gluconeogenesis was the most enriched pathway in this study. GAPDH and NAD binding were identified as “intersection DEPs”. Kanehisa, M., Furumichi, M., Tanabe, M., Sato, Y., Morishima, K. KEGG: new perspectives on genomes, pathways, diseases and drugs. Nucleic Acids Res. 45, D353–D361 (2017).

Gluconeogenesis was the most enriched metabolic pathway across the four developmental stages, resulting in the generation of glucose (C_6_H_12_O_6_) from non-carbohydrate carbon substrates, and is responsible for maintaining normal blood glucose levels in humans and many other animals^24^, while glycolysis converts glucose into pyruvate (CH_3_COCOO-), with the release of free energy used to form the high-energy molecules ATP (adenosine triphosphate) and NADH (reduced nicotinamide adenine dinucleotide)^25^ (Fig. 4). The produced energy is then available for body growth and development. In this study, GAPDHand NAD binding were identified as “intersection DEPs”. The product of GAPDH catalyzes an important energy-yielding step in carbohydrate metabolism through the reversible oxidative phosphorylation of glyceraldehyde-3-phosphate in the presence of inorganic phosphate and NAD^26^. The citric acid cycle was the other main enriched pathway involved in the energy formation. The citric acid cycle, also known as the tricarboxylic acid cycle, involves a series of chemical reactions that release stored energy via oxidation of acetyl-CoA derived from carbohydrates, fats, and proteins into carbon dioxide and chemical energy, in the form of ATP^27^. Aconitate hydratase was an important DEP between neurula and heart-beat stages (1.63) which can cleave carbon-oxygen bonds to release ATP^28^.

**Figure 5:**
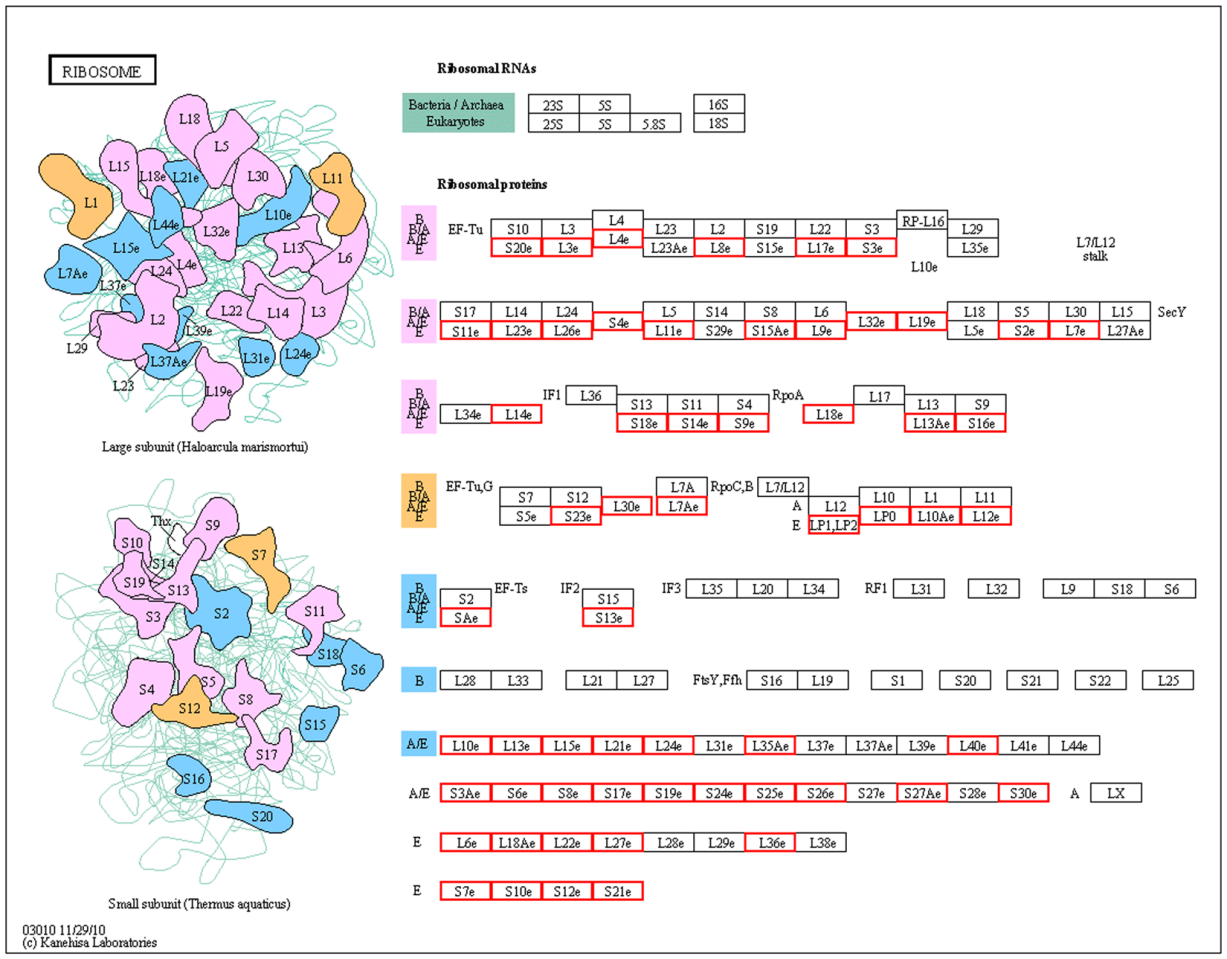
Ribosome metabolic pathway. According to the KEGG analysis of DEPs, Ribosome was the main enriched pathway in this study. The 60S ribosomal proteins L12, L17, and L22 were identified the “intersection proteins”. Kanehisa, M., Furumichi, M., Tanabe, M., Sato, Y., Morishima, K. KEGG: new perspectives on genomes, pathways, diseases and drugs. Nucleic Acids Res. 45, D353–D361 (2017).

The ribosome is other main enriched pathway across the four developmental stages, which is a complex molecular machine found within all living cells, serving as the site of biological protein synthesis (translation)^29^ (Fig. 5). Ribosomes link amino acids together in the order specified by message RNA (mRNA) molecules. Ribosomes can be divided into small 40S and large 60S ribosomal subunits^29^ together composed of four RNA species and approximately 80 structurally distinct proteins. The small ribosomal subunits read the RNA, while the large ribosomal subunits join amino acids to form a polypeptide chain. Each subunit is composed of one or more ribosomal RNA (rRNA) molecules and a variety of ribosomal proteins. In this study, the 60S ribosomal proteins L12, L17, and L22 were identified as “intersection proteins”. These ribosomal proteins are encoded by the *RPL12*, *RPL17*, and *RPL22* genes, respectively, in humans, which each encode a component of the 60S subunit. RPL12 and RPL17 belong to the L11P and L22P families of ribosomal proteins, respectively, located in the cytoplasm, whereas RPL22 belongs to the L22E family of ribosomal proteins.

Other main enriched metabolic pathways included the Proteasome. Proteasomes are protein complexes that mainly function to degrade unneeded or damaged proteins by proteolysis^30^. 26S protease regulatory subunit 6A encoded by PSMC3 gene was considered as an “intersection protein”. Component of the 26S proteasome plays essential roles in the maintenance of protein homeostasis by removing misfolded proteins, damaged proteins and the unnecessary protein. 26S protease regulatory subunit 6A unfolds ubiquitinated targeted proteins, and translocated them into a proteolytic chamber for degradation. The expression level of 26S protease regulatory subunit 6A was up-regulated at blastula stage, compared to that of fertilized egg and neurula, which is consistent with that the hatching rate at blastula stage was the lowest in Amur sturgeon. Further studies are needed to investigate the roles of these proteins^31^.

To the best of our knowledge, although the measurements of microelements have been performed in mature tissues in many aquatic species and even in sturgeon species^32–34^, the measurements during the embryonic developmental stages have not yet been reported in any aquatic species. Only one previous study has performed the microelements measurement aged from 6 to 12 months in 90 aquatic species, results of which indicated that the levels of microelements were significantly influenced by the culture site and culture conditions^35^. Fe deficiency was associated with decreased enzyme activities in fish, and disturbed growth and immunity^36^. The contents of Fe, Mn, and Cu decreased and then increased again across the four embryonic developmental stages in the current study, while the Zn content gradually increased and then decreased. Mn is an important component of several enzymes in animals, including arginine kinase, proline peptidase, RNA polymerase, superoxide dismutase, and pyruvate carboxylase, which have been shown to promote skeleton development, fertilization, hatchability, and feed conversion^37^. In this study, the peak Mn content occurred at the fertilized egg, and then decreased dramatically with the embryonic development, indicating that Mn was mainly obtained from their parents. Cu is involved in the formation of cytochrome oxidase, lysine hydrochloride, superoxide dismutase, and tyrosinase, which play essential roles in the defensive immune system in fish^37–39^. Cu is also involved in regulating the assimilation and utilization of Fe. Cu cannot be synthesized by fish, thus it should be efficiently absorbed from water environment^40^. The peak Cu content occurred during the heart-beat stage in the current study, indicating that the germ cell absorbed Cu from the water environment during embryonic development. Zn is a necessary microelement in fish, with a wide range of physical functions. It is involved in several metabolic processes via alkaline phosphatase, including nucleotide and protein synthesis, energy metabolism, and redox reaction, which can affect substance metabolism, growth, and development in fish^41,42^. In this study, the Zn content was maintained at a high level in all four embryonic stages, indicating that Zn plays vital roles throughout embryonic development in *A. schrenckii*^43^. Compared to the previous measurement of microelements in skin of farmed *A. schrenckii*, the contents of Fe and Cu were significantly higher in this study, while Zn was significantly lower^33^. These results further confirmed that Fe and Cu cannot be synthesized by *A. schrenckii*.

To the best of our knowledge, the measurements of antioxidants during the embryonic developmental stages were not reported in any aquatic species. The measurements of antioxidants were performed under stress condition in sturgeon species, including the changes in food diets and water conditions, in order to analyze the effects of such changes on antioxidant defenses^44–48^. Superoxide dismutase (SOD), Catalase (CAT), Malondialdehyde (MAD), and GSH are important antioxidants in fish, maintaining normal embryonic development through protecting the cytomembrane, DNA, and proteins from damage by reactive oxygen species^49,50^. GSH is involved in maintaining normal immune system functioning, including antioxygenation and antidotal action^51^. SOD is an important antioxidant with essential roles in removing and eliminating the negative effects of free radicals, and SOD content is a vital index indicating senility and death^52–54^. The GSH and SOD levels at heart-beat stage are 10 times and 5 times higher than other stages. These results suggested essential roles of GSH and SOD in immune system, which remove negative effects to ensure the normal hatching of *A. schrenckii* embryos. CAT accounts for about 40% of all expressed peroxidases, and occurs in every tissue, with especially high expression in the liver. CAT can catalyze H_2_O_2_ to H_2_O and O_2_, thus preventing H_2_O_2_ from binding with O_2_ to form hydroxyl radicals, which are very harmful products in biological systems^53–55^. MDA is an important product in the peroxidation reaction of membrane lipids. MDA generation dramatically increases membrane damage leading to cell death, and is thus an important index for the analysis of senility and stress-resistance processes^56^. The level of MDA at each developmental stage was hundred folds higher than that of other antioxidants, while the levels of SOD and CAT across the developmental stages were hundred folds lower than that in mature tissues in other sturgeon^44,45^, indicating the significant presence of MDA and the deficiency of SOD, GSH and CAT may be the main reason leading to a low hatching rate in *A. schrenckii*.

In conclusion, 74, 77, and 76 proteins were differentially expressed between the fertilized egg and blastula, blastula and neurula, and neurula and heart-beat stages of *A. schrenckii* development, respectively, according to iTRAQ analysis. On the basis of GO and pathway enrichment analyses of DEPs, Gluconeogenesis and Citrate cycle were considered to provided energy; Ribosome promoted protein synthesis; and Proteasome protected immune system, in order to maintain normal embryonic development of *A. schrenckii*. Analysis of levels of microelements and antioxidants indicated that Mn, Cu and Fe were obtained from their parents or water environment in *A. schrenckii*, while the dramatically high level of Zn plays vital roles throughout embryonic development. The dramatically high level of MDA across the embryonic development may be the main reason leading to a low hatching rate in *A. schrenckii*.

## Materials and Methods

### Ethics statement

All the fish handling and experimental procedures involved in this study were approved by the Animal Care and Use Committee of the Heilongjiang River Fisheries Research Institute, Chinese Academy of Fishery Sciences, Haerbin, and followed the experimental basic principles. All methods were performed in accordance with relevant guidelines and regulations. All efforts were made under MS222 anesthesia to minimize suffering.

### Sample collection

Three healthy male and three female *A. schrenckii* (body weight 15–25 kg) were obtained from the Amur sturgeon breeding and engineering center, Heilongjiang River Fisheries Research Institute, Chinese Academy of Fishery Sciences. The fish were maintained in aerated freshwater at 14 °C.

A temperature of 16 °C–18 °C was used to hasten spawning in *A. schrenckii*. Female *A. schrenckii* received two injections of luteinizing hormone-releasing hormone A2 at 2 μg/kg and 4 μg/kg 12 h after the first injection, respectively. Male *A. schrenckii* were injected with 1 μg/kg luteinizing hormone-releasing hormone A2 1day prior to the first injection of female *A. schrenckii*. Mature sperm and eggs were obtained, fertilized for 2 min by a semi-dry technique, and debonded for about 30 min using 30% talcum powder. The fertilized eggs were then transferred to an incubator at 18 °C and oxygen level >6.0 mg/L. The fertilized eggs were observed under a microscope and embryos were collected at the fertilized egg, blastula, neurula, and heart-beat stages in micro tubes, and immediately frozen in liquid nitrogen to prevent total protein degradation, until use for protein extraction for proteome profiling analysis.

### Protein preparation and iTRAQ labeling

We analyzed the protein expression profiles of the four embryonic developmental samples of *A. schrenckii*. At least 100 embryos per group were collected and pooled to eliminate the effects of individual differences. Total proteins were extracted from each developmental sample using a phenol extraction procedure, and the protein concentrations were determined using the Bradford colorimetric method. A total of 100 mg of protein from each sample was lyophilized using a speed vacuum system (Martin Christ, Germany).

iTRAQ labeling was performed using an iTRAQ Reagent 8-Plex kit (Applied Biosystems, Foster City, CA, USA) according to the manufacturer’s protocol. A total of 100 mg extracted protein from each sample was dissolved in 0.5 M triethylammonium bicarbonate, incubated with 10 mL 1 mg/ml trypsin (Promega, Madison, WI, USA) at room temperature for 2 h, and labeled using iTRAQ Reagent 8-Plex kit.

### Strong cation exchange chromatography (SCX) fractionation

The iTRAQ-labeled samples were then pooled and purified using a SCX column (Phenomenex, Torrance, CA, USA), followed by separating using a liquid chromatography-20AB (LC-20AB) HPLC system (Shimadzu, Kyoto, Japan). The peptide mixtures were reconstituted with 4 mL buffer A (25 mM NaH_2_PO_4_ in 25% ACN, pH 2.7). The reconstituted peptide mixtures were loaded onto a 4.6 × 250 mm Ultremex SCX column with 5 μm particles (Phenomenex), and eluted at a flow rate of 1 mL/min with elution buffer B (25% v/v acetonitrile, 25 mM NaH_2_PO_4_, 1 M KCl, pH 2.7) for 7 min, 5%–60% buffer B for 20 min, and finally 60%–100% buffer B for 2 min, and maintained for 1 min. The absorbance at 214 nm was monitored and 12 fractions were collected. Samples of each fraction were dried and desalted before LC-electrospray ionization-MS/MS analysis^57^.

### *LC*-*MS*/*MS* analysis

Each dried sample was reconstituted to an average of 0.5 μg/μL using buffer A (5% ACN, 0.1% FA), and followed by centrifuging at 20,000 × *g* for 10 min to remove impurities. A total of 2.5 μg protein was separated using a LC-20AD HPLC system (Shimadzu, kyoto, Japan). A flow rate of 8 μL/min was used to load the sample onto a Trap column at within 4 min, and then transferred to an analysis column at a flow rate of 300 nL/min with elution buffer B (95% ACN, 0.1% FA) for 5 min, 5%–35% buffer B for 35 min. Buffer B was increased to 60% within 5 min, to 80% within 2 min, maintained for 2 min, finally decreased to 5% within 1 min, and maintained for 10 min. The sample was then transferred to a mass spectrometer (Triple TOF 5600; AB SCIEX) for LC-MS/MS analysis, as described previously^20^. A 2.5 kV ion spray voltage, 15 psi nebulizer gas, 30 psi curtain gas, and interface heater temperature of 150 were used to perform the data analysis. A RP of ≥30,000 FWHM was used to operate the MS for TOF MS scan, and 250 ms was required for survey scan for IDA analysis. Thirty products were collected if the ion scans were more than a threshold of 120 counts/s. The total cycle was conducted at 3.3 s. The Q2 transmission window was set at 100 Da for 100%. 40 GHz multichannel TDC detector containing a four-anode channel was used to detect and sum ions from each scan at a 11 kHz pulser frequency value. iTRAQ adjusted rolling collision energy and sweeping collision energy with 35 ± 5 eV were combined for application to all precursors to ensure collision-induced dissociation. Dynamic exclusion was set for 1/2 of peak width (15 s), and the precursor was then refreshed to the exclusion list. MS/MS analysis was used to select peptides with +2 to +3 charge states. The fragment intensity multiplier was set to 20, with a maximum accumulation time of 2 s.

### iTRAQ protein identification and quantification

MASCOT 2.3.02 software was used to analyze iTRAQ data. To conduct confident peptide identification, a Mascot probability analysis was used to count the unique peptides with ratios of significance scores (≥20) at the 99% confidence interval. A protein was identified when it contained at least one unique peptide, while at least two unique peptides needed to be matched to a protein for analysis. The median ratio in Mascot was used to perform the weight and normalization of the quantitative protein ratios. The confidence level of the altered expression of proteins was calculated by Protein Pilot as a p-value. We only used ratios with P-values < 0.05 and a false discovery rate (FDR) < 0.05, and only proteins with fold changes of >1.2 were considered as up-regulated proteins or proteins with label ratio of <0.83 were considered as down-regulated proteins.

### GO and pathway enrichment analysis

Functional protein annotations according to GO were determined using the Blast2GO program. Hypergeometric tests with a ratio of P < 0.05 were used to identify all and elsewhere differentially expressed proteins in the GO enrichment terms. The proteins were further subjected to functional annotation analysis according to the COG (http://www.ncbi.nlm.nih.gov/COG/) and KEGG databases (http://www.genome.jp/kegg/). Pathways that were significantly enriched compared with differential proteins with P < 0.05 and FDR < 0.05 were used as a threshold to select significant KEGG pathways.

### Microelements and antioxidants contents

A total of 1 g*A. schrenckii* embryos at different stages were dry-ashed and digested by 16 ml nitric acid, 2 ml perchilric acid and 1 ml hydrogen peroxide for overnight. The samples were then incubated at 120 °C for 2 h, and incubated at 150 °C until only 2 ml samples were left. The Fe, Mn, Cu, and Zn contents were then measured by AA6800 atomic absorption spectrophotometry (Shimadzu, Japan) according to the GB/T5009. 12(13, 14, 87, 90, 91, 92, 93 and 123)-2003.

Collected different-stage embryos were also diluted in 0.5 ml phosphate-buffered saline, homogenized for 5 min on ice, and centrifuged at 3000 r/min for 10 min. The supernatant was then used for determination of antioxidant contents. The GSH, SOD, MDA, and CAT contents at different embryonic developmental stages were measured using kits from Nanjing Jiancheng Bioengineering Institute, according to the manufacturer’s protocols. The measurements of each microelements and antioxidants level were performed in triplicate.

### Statistical analysis

Quantitative data of the measured microelements and antioxidants level were expressed as mean ± standard deviation using Microsoft Excel 2010. The level of significance between different microelements and between different antioxidants was analyzed by one-way analysis of variance (ANOVA) and post hoc Duncan multiple range tests with SPSS software version 17.0. Statistically significant differences were examined by paired t-test. A probability level of 0.05 was considered to be statistically significant (P < 0.05).

## Electronic supplementary material

Supplementary information 1

Supplementary information 2

Supplementary information 3
